# Fecal microbiome of growing pigs fed a cereal based diet including chicory (*Cichorium intybus* L.) or ribwort (*Plantago lanceolata* L.) forage

**DOI:** 10.1186/s40104-015-0054-8

**Published:** 2015-12-18

**Authors:** Johan Dicksved, Janet K. Jansson, Jan Erik Lindberg

**Affiliations:** Department of Animal Nutrition and Management, Swedish University of Agricultural Sciences, P.O. Box 7024, SE75007 Uppsala, Sweden; Department of Microbiology, Swedish University of Agricultural Sciences, P.O. Box 7025, SE75007 Uppsala, Sweden; Division of Biology Earth and Biological Sciences, Pacific Northwest National Laboratories, P.O. Box 999, MSIN J4-18, WA99352 Richland, WA USA

**Keywords:** Amplicon sequencing, Chicory, Microbiome, Ribwort, Uronic acid, Weaning, 16S

## Abstract

**Background:**

The purpose of this study was to investigate how inclusion of chicory forage or ribwort forage in a cereal-based diet influenced the fecal microbial community (microbiome) in newly weaned (35 days of age) piglets. The piglets were fed a cereal-based diet without (B) and with inclusion (80 and 160 g/kg air-dry forage) of vegetative shoots of chicory (C) and leaves of ribwort (R) forage in a 35-day growth trial. Fecal samples were collected at the start (D0), 17 (D17) and 35 (D35) days after weaning and profiles of the microbial consortia were generated using terminal restriction fragment length polymorphism (T-RFLP). 454-FLX pyrosequencing of 16S rRNA gene amplicons was used to analyze the microbial composition in a subset of the samples already analyzed with T-RFLP.

**Results:**

The microbial clustering pattern was primarily dependent on age of the pigs, but diet effects could also be observed. Lactobacilli and enterobacteria were more abundant at D0, whereas the genera *Streptococcus*, *Treponema*, *Clostridium*, *Clostridiaceae1* and *Coprococcus* were present in higher abundances at D35. Pigs fed ribwort had an increased abundance of sequences classified as *Treponema* and a reduction in lactobacilli. However, the abundance of *Prevotellaceae* increased with age in on both the chicory and the ribwort diet. Moreover, there were significant correlations between the abundance of *Bacteroides* and the digested amount of galactose, uronic acids and total non-starch polysaccharides, and between the abundance of *Bacteroidales* and the digested amount of xylose.

**Conclusion:**

This study demonstrated that both chicory and ribwort inclusion in the diet of newly weaned pigs influenced the composition of the fecal microbiota and that digestion of specific dietary components was correlated with species composition of the microbiota. Moreover, this study showed that the gut will be exposed to a dramatic shift in the microbial community structure several weeks after weaning.

## Background

In order to maintain normal physiological functions in the digestive tract of pigs, a minimum level of fiber has to be included in the diet [1]. Moreover, by increasing the fiber level in the diet of weaned piglets, the pH in the hindgut is reduced [2] and the content of organic acids in the stomach and the ileum is increased [2, 3]. These changes in the gut environment, induced by fiber inclusion, indicates a shift in dominating bacterial population which may impair the conditions for pathogenic bacteria and may be more beneficial for maintaining gut health [1, 4]. Fiber properties (soluble vs. insoluble) and age of the pig will modulate the impact of fiber level on the gut environment [2]. Soluble fiber is well digested by both growing pigs and sows, whereas sows have a higher capacity to digest insoluble fiber [5].

Chicory (*Cichorium intybus* L.) and ribwort (*Plantago lanceolata* L.) are dicotelydenous herbs with a high content of uronic acid (80–90 g per kg dry matter) of which approximately 80 % is soluble. Uronic acid in dicotelydenous plants derives from galactosyluronic acid that is the building block in pectins [6]. Uronic acid has a high digestibility in forage crops fed to growing pigs [7, 8]. Moreover, pectin substances from sugar beet pulp have been shown to influence the gut microbial ecosystem, in particular by increasing the fecal *Lactobacillus* counts [9], and is therefore a very interesting fiber component in piglet nutrition.

We have previously shown that inclusion of chicory in the diet influenced the intestinal micro-environment and the microbiota in pigs [10–12]. For example, inclusion of chicory forage was associated with higher abundance of ileal lactobacilli and colonic butyrate producing bacteria [11]. In addition, chicory forage inclusion influenced the relative abundance of *Prevotella*, but the change in abundance was dependent on species of *Prevotella* [10, 12]. We also found correlations between specific bacterial groups and short chain fatty acid (SCFA) profiles, which shows that inclusion of chicory is influencing the intestinal micro-environment. However, less is known about ribwort inclusion and its influence on the microbiota. Ribwort forage contains a range of bioactive and antimicrobial compounds that may influence the microbiota [13].

The recent technological development of the Next Generation Sequencing platforms has facilitated a deeper analysis of the gut microbiota composition, more recently referred to as “microbiome”. The aim of this experiment was therefore to characterize the post weaning gut microbiome and to get a deeper understanding of how inclusion of chicory and ribwort forage in a cereal-based diet influences the microbiome in weaned piglets. Furthermore, we aimed to identify correlations between dietary components and the composition of the intestinal microbiome.

## Methods

### Experimental setup

The study included 19 5-wk old weaned and castrated male piglets (Swedish Landrace × Yorkshire) used in a growth trial. The pigs originated from five different litters and had a live weight of 11.7 kg (s.d. 0.8 kg) at the start of the experiment. The piglets were purchased from a herd free from diseases according to the A-list of International Office of Epizootics [14] and were housed individually in pens equipped with a rubber mat, urine drainage and no bedding.

Piglets had ad libitum access to feed and water throughout the experiment, except for the first days when the feed allowance was restricted. The experimental diets comprised a cereal-based basal diet (B) and two diets composed to contain 80 and 160 g/kg air-dry forage made from vegetative shoots of chicory (C80, C160) and leaves of ribwort (R80, R160), respectively. The basal diet was composed of ground cereals (wheat, barley and oats), milled through a 5-mm screen, supplemented with protein, amino acids, mineral and vitamins to meet nutritional requirements of piglets (Table 1). In diets with chicory and ribwort inclusion, the cereal mixture was substituted with the herbs on an air-dry basis (Table 1). The herbs where harvested at the vegetative stage (September) with a stubble height of *c*. 5 cm and dried with forced air at 30 °C for a week and milled through a 5-mm screen before mixing with the other feed ingredients.

**Table 1:** Ingredient composition (g/kg) of the experimental diets

Ingredients	Basal	Chicory forage		Ribwort forage	
		80	160	80	160
Wheat	400	360	320	360	320
Barley	300	270	240	270	240
Oat	100	90	80	90	80
Chicory forage	-	80	160	-	-
Ribwort forage	-	-	-	80	160
Fish meal	30	30	30	30	30
Potato protein	50	50	50	50	50
Casein	40	40	40	40	40
Sugar	40	40	40	40	40
Vitamin–mineral premix^1^	11	11	11	11	11
Others^2^	29	29	29	29	29

^1^Content/kg premix: vitamins (mg): A 1,000,000 IE, D 100,000 IE, E 6,000, K3 200, B1 200, B2 400, B6 300, B12 2, panthothenic acid 1,500, niacin 2,000, biotin 25. Minerals (mg): Fe 4,000, Cu 1,000, Mn 2,000, Zn 7,000, I 30, Se 35. ^2^Content/kg others: lysine 1.2, methionine 0.08, threonine 0.03, dibasic-calcium phosphate 20, Ca-carbonate 2.5, NaCl 2.5, FeSO_4_ + 4H_2_O 0.24, titanium (IV) oxide 2.5

The experiment was organized according to a randomized block design, with three replicates for the low inclusion level of forage, four replicates for the highest inclusion level of forage and five replicates for basal diet (Table 2). Fecal samples were collected at the start of the experiment (D 0), after 17 d (D 17) and after 35 d (D35).

**Table 2:** Performance of weaned piglets and indication of samples used for T-RFLP and 454-pyrosequencing analysis

Items	Number	Weight gain, g/day	Feed intake, g/day	T-RFLP (D0:D17:D35)	454-Seq. (D0:D17:D35)
Control	5	690^a^	1135^a^	5:5:5	3:0:3
Chicory 80	3	657^a^	1181^a^	3:3:3	0:0:0
Chicory 160	4	629^a^	1143^a^	4:4:4	3:0:3
Ribwort 80	3	640^a^	1101^a^	3:3:3	0:0:0
Ribwort 160	4	528^b^	970^b^	4:4:4	3:0:3

Different letters within column indicates a significant difference at *P* < 0.05

The experiment was carried out at the Swedish University of Agricultural Sciences (SLU) and was approved by the ethical committee of the Uppsala region.

### Chemical composition and digestibility of diets

Chemical composition of the experimental diets (Table 3) as well as the digestibility of the dietary components had been characterized previously [7]. The chemical analysis included quantification of total soluble and insoluble non-starch polysaccharides (NSP) and their constituent sugars including uronic acids. The digested amount of arabinose, xylose, mannose, galactose, glucose, uronic acids, and total NSP was based on the intake and digestibility of each component.

**Table 3:** Chemical composition (g/kg dry matter) of basal diet, chicory forage and ribwort forage

Items	Basal diet (B)	Chicory forage (C)	Ribwort forage (R)
Ash	67.0	255.6	133.6
Crude protein	211.4	195.2	169.4
Crude fat	26.9	15.6	11.1
Starch	463.0	24.5	9.5
WSC	60.5	11.5	42.0
NDF	107.6	268.0	352.0
NSP			
*Total*	187.2	311.0	308.5
*Insoluble*	142.6	194.9	229.5
Glucose			
*Total*	89.4	133.7	134.9
*Insoluble*	56.9	118.3	134.9
Arabinose			
*Total*	25.0	13.4	17.8
*Insoluble*	22.4	5.6	10.4
Xylose			
*Total*	56.7	31.1	32.1
*Insoluble*	56.2	27.2	26.8
Uronic acid			
*Total*	7.4	97.0	88.3
*Insoluble*	4.0	21.2	19.9
Klason Lignin	34.9	107.2	77.7
Dietary fibre	222.0	418.4	386.2

Abbreviations: *WSC* water soluble carbohydrates (free glucose + free fructose + sucrose + fructan), *NDF* neutral detergent fibre, *NSP* non-starch polysaccharides

### Terminal-restriction fragment length polymorphism (T-RFLP) analyses

DNA was isolated from fecal samples in triplicates according to the method described by Leser et al. 2002 [15]. The 16S rRNA genes were PCR amplified from each DNA extract using the general bacterial primers Bact-8 F (5′-AGAGTTTGATCCTGGCTCAG-3′) [16], 5′ end-labeled with 6-carboxyfluorescein (6-FAM), and 926r (5′-CCGTCAATTCCTTTRAGTTT-3′) [17] under conditions described elsewhere [18]. DNA product amounts and sizes were confirmed by agarose gel electrophoresis using GeneRuler 100 bp DNA ladder Plus (Fermentas Life Sciences, Burlington, Canada) as a size marker.

PCR products were digested with restriction enzyme *Hae*III and the resulting fragments were separated on an ABI 3700 capillary sequencer (Applied Biosystems, Foster City, CA). The sizes of the fluorescently labelled fragments were determined by comparison with the internal GS ROX-500 size standard (Applied Biosystems). The T-RFLP electropherograms were imaged using the Peak scanner software (Applied Biosystems) and relative peak areas of each terminal restriction fragment (TRF) were determined by dividing the area of the peak of interest by the total area of peaks, using 50 and 500 bp lower and upper threshold values, respectively. Data was normalized by applying a threshold value for relative abundance at 0.5 %, and only TRFs with higher relative abundances were included in the remaining analyses.

### 454-pyrosequencing analysis

The pig fecal microbiome was characterized with higher resolution in a subset of the pigs by 454-pyrosequencing (Table 2). The (V5 and V6) variable regions of the 16S rRNA gene were amplified by PCR using forward primer (784f 5′- AGGATTAGATACCCTGGTA 3′) and reverse primer (1061r 5′ CRRCACGAGCTGACGAC 3′). The reverse primer was tagged with 1 of 4 labels (CGAT, CATG, CTGA and CGTA) at the 5′ end along with the adaptor sequence (5′- GCCTCCCTCGCGCCATCAG 3′) to allow 4 samples to be included in a single 454-FLX pyrosequencing lane as previously described [19]. Two microliters of DNA was added to each 25 μL PCR reaction containing 2.5 μL 10 × PCR buffer (Amersham Biosciences, Piscataway, NJ), 1 μL BSA (10 mg/mL) (Amersham Biosciences), 1 μL dNTP (5 mmol/L), 0.25 μL *Taq Polymerase* (5 U / μL) (Amersham Biosciences) and 1 μL of each primer (10 μmol/L) (Scandinavian Gene Synthesis, Köping, Sweden). PCR reactions were carried out on a GeneAmp (Applied Biosystems, Foster City, CA) PCR system (5 min at 94 °C, 30 cycles of 94 °C for 45 s, 55 °C for 40 s and 72 for 1 min, and a final extension of 72 °C for 7 min). Triplicate PCRs were pooled and 60 μL were run on 1 % agarose gels at 80 V for 1.5 h. PCR products of the appropriate size (Approx. 340 bp) were gel purified (QIAquick Gel Extraction Kit, Qiagen, Gmbh, Germany) and eluted in 50 μL of elution buffer. DNA quality was assessed on a Bioanalyzer 2100 (Agilent Technologies, Santa Clara, CA). DNA concentration was measured on a NanoDrop ND-1000 (NanoDrop Technologies, Wilmington, DE) and 25 ng of four samples, labeled with different tag sequences, were pooled and diluted in water for a total of 100 ng in 10 μL. Pyrosequencing was performed on a 454 Life Sciences Genome Sequencer FLX machine (Roche), at the Swedish Institute for Infectious Disease Control, Solna, Sweden.

### Taxonomic analysis

Sequences were checked for quality and sequences that were less than 200 bp in length, that contained incorrect primer sequences, or that contained more than 1 ambiguous base were discarded. Assignment of sequences to samples was based on the 4-bp barcode. Remaining sequences were then subjected to complete linkage clustering using the pyrosequencing pipeline at RDP-X using a conservative 5 % dissimilarity to define operational taxonomic units (OTUs) because of the short sequence length. The most abundant sequence from each OTU was selected as a representative sequence and was taxonomically classified by BLAST searching against a local BLAST database comprised of 269,420 bacterial 16S rRNA gene sequences longer than 1,200 bp with good Pintail scores from RDP v. 10.7. The OTU inherited the taxonomy (down to genus level) of the best scoring RDP hit fulfilling the criteria of ≥ 95 % identity over an alignment of length ≥ 180 bp.

### Statistical analysis

To visualize time or diet related effects in composition of the microbiota, relative abundance values and sizes of T-RFLP fragments were analyzed with principal component analysis (PCA) using the software Canoco (version 4.5, Microcomputer Power Ithaca, NY, USA). For the 454 data, principal coordinate analysis (PCoA) based on Bray Curtis distances were used to monitor clustering pattern of the microbial architecture using the software PAST [20]. To identify specific taxa that correlated with diet or time, statistical analyses were performed using GLM in SAS (SAS Institute, Cary, NC, USA, version 9.1). Pearson correlation analysis was used to identify correlations between digested amount of dietary components and the abundance of microbial taxa. The level of significance was set at *P* < 0.05 and the Benjamini and Hochberg method was used to account for multiple comparisons, based on global *P* values of the variables compared [21].

## Results

### Pig performance

Herb inclusion affected (*P* < 0.05) the average daily feed intake during the experiment (day 0–35; Table 2), with lower intake for the diet (R160) with the highest ribwort inclusion than for the other diets [7]. Inclusion of chicory did however not impair feed intake compared with the basal diet. Moreover, as a consequence of the lower feed intake on the diet with the highest inclusion of ribwort, the daily weight gain was lower (*P* < 0.05) than for the other diets [7]. There was no negative impact on the daily weight gain of including chicory in the diet.

### T-RFLP analysis of the fecal microbiome

**Fig. 1:**
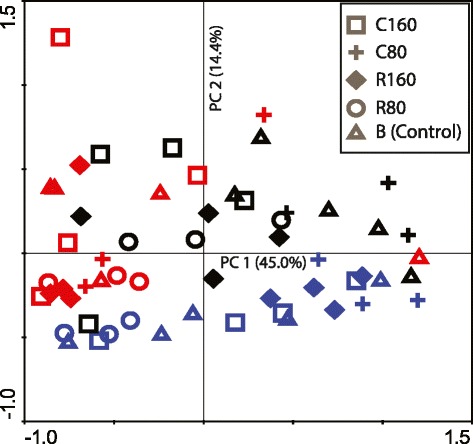
Principal component analysis of Terminal restriction fragment length polymorphism data generated from the fecal microbiome. Symbols colored in blue, represents samples collected at weaning (D 0), in black, samples collected 17 d post weaning (D 17) and red, samples collected 35 days post weaning (D 35). Different symbols represent the different feed supplements, C; Chicory, R; Ribwort. Percentage values represent how much of the variation in data explained by each principal component (PC)

Profiles of the microbial consortia in fecal samples were generated using T-RFLP. Principal component analysis was used to identify patterns in the microbiome that could be explained by factors such as time or diet effects. The PCA showed a clustering pattern that largely was dependent on age of the pigs (Fig. 1). All samples collected at weaning (D 0) clustered separately from the samples collected at D 17 and D 35. There was a large variation in microbial composition between individual pigs at weaning, and the samples were subsequently spread out along the first principal component (PC 1). No apparent clustering pattern of diet effects could be visualized in the PCA scatter plots (Fig. 1).

### Barcoded 454 pyrosequencing analysis of the fecal microbiome

Pyrosequencing of 16S rRNA gene amplicons was used to analyze the microbial composition in a subset of the samples that had already been analyzed with T-RFLP for a more detailed view of the microbial composition. 16S data was obtained from nine pigs from samples collected both at D 0 and D 35 (Table 2). After quality filtering, 31,620 sequences were obtained, with an average of 1,757 sequences per sample (range 1,386–2,095). Analysis of the sequence data revealed a large individual variation between pigs but also that the fecal microbiome at weaning and 35 days after weaning was dominated by the same main phyla, primarily members of the *Firmicutes* (F) and *Bacteroidetes* (B) phyla. These were mainly dominated by the *Lachnospiraceae* (F), *Ruminococcaceae* (F), *Lactobacillaceae* (F), *Streptococcaceae* (F) and *Prevotellaceae* (B) families. In addition, a large fraction of the sequences could not be matched to the sequences in the public databases indicating presence of undescribed species.

### Development of the post weaning microbiome

**Fig. 2:**
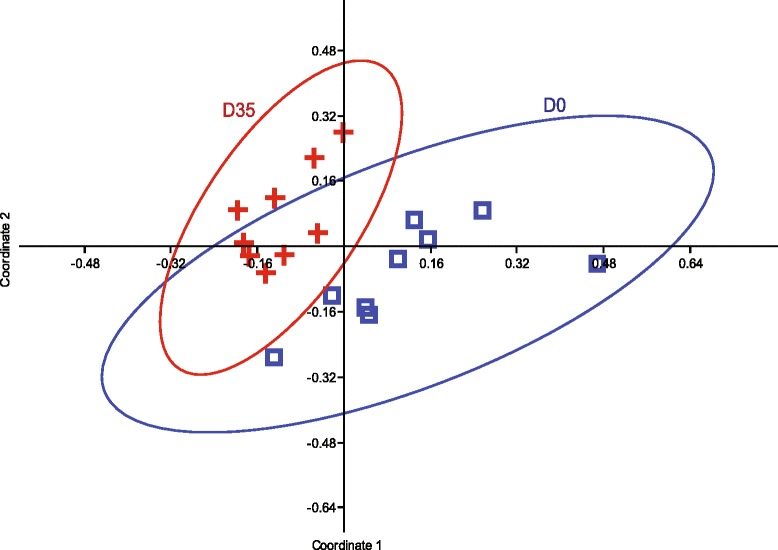
Multivariate analysis of the 16S sequence data. Principal coordinate analysis (PCoA) plot using Bray Curtis distances on the 16S sequence data classified down to genus level. Symbols colored in blue, represent samples collected at weaning (D 0) and red, samples collected 35 days post weaning (D 35)

**Fig. 3:**
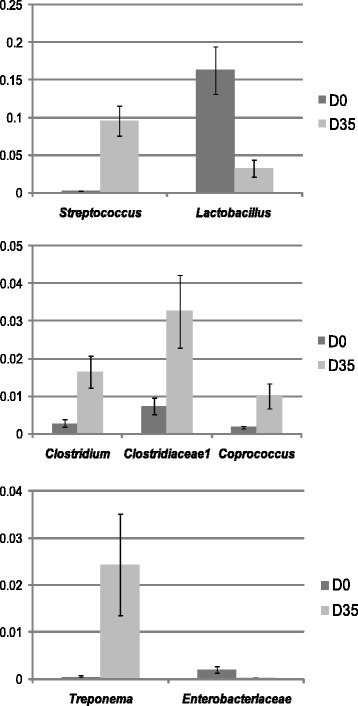
Development of the post weaning microbiome. Barcharts illustrating the taxa that differs significantly in relative abundance between samples collected at weaning (D 0) and 35 days post weaning (D 35)

Principal coordinate analysis (PCoA) based on Bray Curtis distance metrics was used to visualize clustering patterns in the 16S data. The samples arranged into two clusters and in agreement with the T-RFLP data analysis the segregation of samples were associated with the age of the pigs (Fig. 2). Comparing samples at D 0 and D 35 revealed a maturation of the gut microbiota with dramatic changes in the relative abundance of certain genera. Lactobacilli and enterobacteria were more abundant in younger pigs (*P* < 0.05), whereas the genera *Streptococcus*, *Treponema*, *Clostridium*, *Clostridiaceae1* and *Coprococcus* were present in higher abundances in older pigs (*P* < 0.05; Fig. 3). In agreement with the T-RFLP data analysis, the 16S data showed that individual pig samples collected at D 0 had a larger variation in the community structure than the samples collected at D 35 (Fig. 2).

### Diet dependent influences on the microbiome

**Fig. 4:**
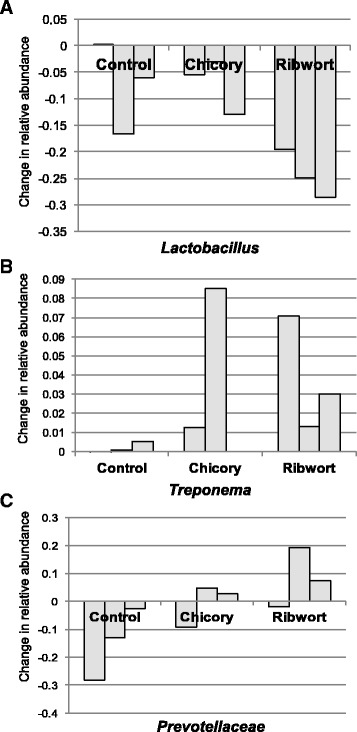
Dietary fiber influence on the microbiome. The bar charts shows bacterial taxa that were influenced by the supplement of chicory or ribwort. Bars illustrating the change in relative abundance between samples collected at weaning (D 0) and 35 days post weaning (D 35) for (**a**) *Lactobacillus,* (**b**) *Treponema *and (**c**) *Prevotellaceae*

Despite the large temporal variation of the developing microbiota it was possible to identify changes that could be linked to inclusion of chicory or ribwort. As indicated earlier, the abundance of lactobacilli was reduced in samples collected at D 35. The reduction of lactobacilli was however significantly more pronounced in pigs fed the ribwort diet (*P* = 0.004; Fig. 4). Instead the pigs fed ribwort had a significantly increased abundance of sequences classified as *Treponema* (*P* = 0.011; Fig. 4). Another microbial group that was associated with the diet regime was *Prevotellaceae*. The abundance of *Prevotellaceae* decreased in animals fed the control diet whereas it increased in animals fed both the chicory and the ribwort diet (*P* < 0.001; Fig. 4).

### Correlations between digestion of dietary components and specific groups of microbes

Pearson correlations were tested between abundance data from all microbial taxa from samples collected at D 35 and the digested amount of arabinose, xylose, mannose, galactose, glucose, uronic acid and total NSP from the same samples. We found a significant correlation between the abundance of *Bacteroides* and the digested amount of galactose and uronic acids (Table 4). In addition, the abundance of *Bacteroides* was also positively correlated with the digested amount of NSP, but the correlation did not reach significance (Table 4). Furthermore, the abundance of *Bacteroidales* was positively correlated with the digested amount of xylose (Table 4).

**Table 4:** Pearson correlations between bacterial abundance data and digested amount of dietary compounds

Variable	With variable	r	*p*-value
*Bacteroides*	galactose	0.745	0.021
*Bacteroides*	uronic acid	0.688	0.041
*Bacteroides*	NSP	0.648	0.059
*Bacteroidales*	xylose	0.678	0.045

Abbreviations: *NSP* non-starch polysaccharides

## Discussion

The fecal microbiome changed dramatically in composition after weaning, regardless of diet, but inclusion of the different fiber sources had an impact on the development of the post weaning microbiome. Inclusion of ribwort had a larger effect on the post weaning microbiome compared to chicory (Fig. 4). The abundance of lactobacilli was lower in samples collected at weaning (D 0) compared with samples collected 35 days post weaning (Fig. 3). However, for pigs fed the diet including ribwort, the abundance of lactobacilli had decreased to a larger extent compared with the other diets (Fig. 4). This indicates that ribwort inclusion has a negative impact on lactobacilli in the gut. Both chicory and ribwort have a high content of uronic acids, which derives from galactosyluronic acid and is a building block in pectins [6]. Pectin of plant origin, such as sugar beet pulp, has been used as a fibrous feedstuff in pig diets and resulted in an increased lactic acid bacteria (LAB) population in the small intestine [22]. In addition, we have earlier shown that inclusion of chicory forage was associated with higher abundances of LAB, primarily in ileal digesta, to a lesser extent in colonic digesta [11] but not in fecal samples [10]. In the present study, the chicory feed did not impact the fecal lactobacilli compared with the control. It is therefore likely that the effect of chicory forage on lactobacilli occurs primarily in the small bowel.

Our study showed that the abundance of *Prevotella* increased in pigs fed the chicory and ribwort diets compared to the control feed. *Prevotella* is one of the abundant bacteria found in the pig gut. This group of gram-negative bacteria is able to produce several xylanases, mannanases, β-glucanases, and corresponds to soluble xylan utilization, and is therefore likely important for biodegradation of complex sugars in the gut [23]. Rural African children and rural Papua New Guinea habitants, living on a fiber-rich diet harbor a gut microbiota rich in *Prevotella* spp. while this community is less abundant in European children and habitants in the United States, living on a ‘Western’ diet (typically high in animal protein, sugar, starch, and fat and low in fiber) [24, 25]. This indicates that the abundance of *Prevotella* is influenced by the fiber content in the diet but the type of fiber is also important. For example, it was shown that ruminal *Prevotella ruminicola* and *Prevotella bryanti* responded in opposite directions to hay and grain-based diets [26].

In the current dataset, the abundance of *Bacteroides* was positively correlated with the digested amount of galactose and uronic acid. In addition, the abundance of sequences classified as *Bacteroidales* was correlated with the digested amount of xylose. *Bacteroides* and *Prevotella* are the major carbohydrate degrading organisms in the gut and it is therefore not surprising that these positive correlations were found. Uronic acid is extensively fermented in the colon, but utilization of uronic acid is restricted to few genera. *Bacteroides* have the ability to utilize uronic acid [27, 28] as too do *Faecalibacterium* [11, 29]. We could, however, not find a significant correlation between the abundance of *Faecalibacterium* and the digested amount of uronic acid.

The pigs fed the highest inclusion of ribwort had a significantly lower feed intake and weight gain compared with pigs fed the other diets. However, it is not known if the reduced weight gain and feed intake influenced the microbiome. Neither is it possible to conclude to what extent the dietary influence in the microbiota structure was masked by the natural change in microbiota structure after weaning. The development of the post weaning microbiome was characterized by a dramatic shift in the bacterial composition with a marked reduction of lactobacilli and *Enterobacteriaceae*, and an increased abundance of the genera *Streptococcus*, *Clostridium*, *Clostridiaceae1*, *Treponema*, and *Coprococcus*. These bacterial groups are commonly detected in weaned pigs reflecting that the fecal microbiome in the pigs included in this study has a composition similar to what others have shown [30–32]. The dramatic change in the microbiome during weaning is in agreement with earlier studies in human infants [33, 34], and in previous studies in pigs that have shown a shift from a *Lactobacillus* dominated microbial population towards dominance of *Streptococcus* [35, 36]. In addition, the reduction in relative abundance of *Enterobactericeae* with increasing age was in agreement with earlier culturing data from the same animals [7].

## Conclusion

In conclusion, this study demonstrated that both chicory and ribwort inclusion as feed supplements in the diet of newly weaned pigs, influenced the composition of the fecal microbiome. The feed supplements were associated with a change in the abundance of *Lactobacillus*, *Treponema* and *Prevotella*. Furthermore, we showed that digestion of specific dietary components was correlated with the species composition of the microbiota. However, the most dramatic change in the microbiota was found when fecal samples collected 17 and 35 days post weaning were compared with samples collected at weaning and demonstrated that the gut will be exposed to a dramatic shift in the microbial community structure several weeks after weaning.
